# Causes of Condemnations of Edible Parts of Slaughtered Pigs in Bavaria and Their Economic Implications: A Retrospective Survey (2021–2022)

**DOI:** 10.3390/vetsci12020088

**Published:** 2025-01-23

**Authors:** Sebastian Ciui, Adriana Morar, Viorel Herman, Emil Tîrziu, Mirela Imre, Alexandra Ban-Cucerzan, Sebastian Alexandru Popa, Răzvan-Tudor Pătrînjan, Doru Morar, Kálmán Imre

**Affiliations:** Faculty of Veterinary Medicine, University of Life Sciences “King Mihai I” from Timişoara, 300645 Timișoara, Romania; sebastian.ciui@usvt.ro (S.C.); viorel.herman@fmvt.ro (V.H.); emiltirziu@usvt.ro (E.T.); alexandracucerzan@usvt.ro (A.B.-C.); sebastian.popa@usvt.ro (S.A.P.); razvan.patrinjan.fmv@usvt.ro (R.-T.P.); dorumorar@usvt.ro (D.M.); kalmanimre@usvt.ro (K.I.)

**Keywords:** pig, slaughterhouse, postmortem, lesions, economic loss

## Abstract

This study aimed to provide insights into the main causes of postmortem carcass and organ condemnations in a pig slaughterhouse and evaluate the resulting financial impact of the rejection of edible parts of animals. A population of 307,866 pigs, admitted for slaughter between January 2021 and December 2022 in a Bavarian abattoir, was studied. Regarding the examined carcasses, 2.09% (*n* = 6422) presented pathological conditions. Of these, 8.12% (*n* = 522) and 91.88% (*n* = 5900) were totally and partially confiscated, respectively. The main reason for the complete rejection of carcasses was the occurrence of generalized diseases (62.6%), while the presence of abscesses and traumatic and/or tail lesions was the most frequently implicated (70.7%) in partial condemnations. In the case of the organs, 17.59% (*n* = 54,145) presented abnormalities, with distributions of 14.71% (*n* = 45,290), 1.94% (*n* = 5968), 0.72% (*n* = 2213), and 0.22% (*n* = 674) within the examined liver, lung, kidney, and heart specimens, respectively. The principal reasons for liver, lung, kidney, and heart condemnations were the occurrence of parasitic diseases (53.4%), bacterial/viral infections (63.5%), dystrophies/anomalies (98.4%), and bacterial/viral infections (98.5%), respectively. The total financial loss attributable to carcass and organ condemnations was estimated at EUR 392,744.2, which represents 0.40% of the total achievable net revenue without rejections. Of this, EUR 197,120 (0.20%) and EUR 195,624.2 (6.95%) were related to carcass and offal seizures, respectively. The study results offer useful information for veterinarians, stockowners, and epidemiologists to make and set up policies to increase the efficiency and benefits of the swine production system and protect public health.

## Simple Summary

The postmortem inspection of slaughtered animals is viewed as one of the most important tools for monitoring and ensuring that meat is free from communicable diseases. The present study investigated the main causes of carcass and organ condemnations and the resulting economic implications within a population of 307,866 slaughtered pigs. The recorded results showed that 2.09% (*n* = 6422) of the examined carcasses expressed pathological conditions, and out of them, 8.12% (*n* = 522) and 91.88% were totally and partially confiscated, respectively. In the case of the organs, 17.59% (*n* = 54,145) presented abnormalities, with distributions of 14.71% (*n* = 45,290), 1.94% (*n* = 5968), 0.72% (*n* = 2213), and 0.22% (*n* = 674) within the examined liver, lung, kidney, and heart specimens, respectively. The recorded total financial losses attributable to carcass and organ condemnations were EUR 197,120 and EUR 195,624.2, respectively, representing 0.40% of the total achievable net revenue without rejections. This study offers useful information for veterinarians, public health authorities, and pig meat producers.

## 1. Introduction

The rapid speed of global population growth, which has increased over the last few centuries from 1 billion in 1800 to more than 8.1 billion today, is associated with concerns and challenges in ensuring animal-origin protein requirements are met. In this regard, sustainable livestock production, including pig farming, constitutes a major contributor to the maintenance of and improvement in global food security and nutrition in terms of highly nutritious animal-origin foodstuffs [1,2,3].

Pork constitutes the main object of meat consumption, especially in the East Asian region, North America, and Europe. Overall, the pork production of the European Union (EU) member states is the world’s second largest (~23 million tons, representing 21% of the total worldwide production) after China and the first in terms of export capacity (nearly 4 million tons/year). The main producers of pork among EU countries are Spain, Germany, and France, with pork production in these countries representing half of the total EU production and serving as a fundamental component of the food supply and national economies of these countries [4].

Ensuring freshness, safety, and hygiene, as the most important attributes of meat intended for human consumption, is regarded as a fundamental issue for producers, distributors, and consumers [5,6,7]. In this regard, animal health control at the slaughterhouse level as a means of surveillance by veterinary authorities plays a key role in the production of high-quality animal-origin foodstuffs [8]. This action requires significant human and financial resources [9]. Slaughterhouses are considered establishments where meat-producing animals destined for human consumption are slaughtered and controlled within several successive and complementary stages as follows: the regular checking of animal documentation for slaughtering, antemortem inspection, postmortem inspection, and receiving feedback information for the knowledge of disease occurrence and control, especially under subclinical conditions, at the primary production stage [10,11,12]. The successful implementation of these essential procedures, starting from the entry of animals into slaughtering units to obtaining meat, can be viewed as an important defensive barrier against the transmission of zoonotic foodborne diseases to consumers, and it helps to identify animal diseases [2]. Furthermore, during these actions, veterinary inspectors must (i) ensure the optimal health status of animals intended for slaughter based on the principles of animal welfare, (ii) identify and condemn unfit animals for slaughter, (iii) guarantee the hygiene and safety of meat after postmortem inspection, and (iv) implement and maintain proper hygienic and preventive measures within the entire slaughtering process [2,12,13].

However, out of the aforementioned issues, the postmortem inspection of the resulting carcasses and viscera by an official veterinarian is considered a fundamental control point, being the most important tool in monitoring and ensuring that meat is free from communicable diseases [13]. Firstly, the postmortem inspection findings offer useful insights for the evaluation of the efficiency of the implementation of prophylaxis programs at the primary production stage, including biosecurity issues and the effectiveness of disease eradication strategies [11]. In addition, the results can offer feedback on individual data points on animal welfare related to the transport and slaughtering process. Subsequently, the results of the total and partial condemnations provide important data for evaluating and limiting economic losses [2,14].

The available scientific publications focusing on the evaluation of causes of postmortem carcass and organ condemnations, together with economic loss assessment, are still limited and mostly out of data [8,13,15,16]. The available reports highlight a variable prevalence value for the pathologic agents, with a variable financial impact from one study to another, strongly related to the efficiency of disease eradication programs in the primary production stage. Discrepancies in the causes and prevalence of carcass and offal condemnation, reported in various surveys, may be the influence of several factors including climatic conditions of the geographical regions considered, farming procedures and livestock health status, or professional skills of butchers with reduced exposure to human error. Considering the above-mentioned issues, the present investigation was undertaken to contribute to the knowledge of the main causes of carcass and organ condemnations, based on routine meat inspection procedures, and estimate the associated economic impact in a pig slaughterhouse.

## 2. Materials and Methods

### 2.1. Study Design

The investigation was conducted in a private slaughterhouse over two years, enrolling a total of 307,866 slaughtered pigs, between January 2021 and December 2022. The slaughtering unit is located in the southeast of the Federal Republic of Germany and collects pigs from the entire region of Bavaria State (~70,550 km^2^), with an estimated 2.2 million pigs housed in 3130 pig-keeping farms, which represent about 12% of Germany’s pig population [17].

The abattoir, with a slaughtering capacity of around 650–700 pigs per day, collects animals to be slaughtered from 95% industrialized (up to 2000 heads) and another 5% from small and medium-sized (up to 50–80 heads) integrated retail production units. The mapping of the slaughterhouse location was not allowed due to confidentiality terms.

### 2.2. Meat Inspection and Data Collection

After the pig arrives at the slaughtering unit, the sanitary veterinary control activities of the entire process until the meat is obtained were carried out by an adequately trained official veterinary inspector (OVI) according to the stipulated procedures within Official Controls Regulation (EU) no. 2017/625 and Commission Implementing Regulation (EU) no. 2019/627 [18,19].

In the first step, an analysis of the documentation that accompanied the animals to the slaughterhouse (e.g., animal identification cards, health certificate, official movement form, or food chain information form) was performed, followed by an antemortem inspection consisting of a series of procedures and tests, through which the OVI ensured that live animals are safe and suitable for human consumption, without any condition that might negatively influence public and animal health [20]. Next, the entire slaughter process was supervised by the OVI to ensure the fulfillment of the hygienic conditions in agreement with the good manufacturing practice requirements [21]. Subsequently, postmortem inspection, having as the main goal the prevention of epizootic and zoonotic disease transmission to animals and humans, respectively, was achieved after the evisceration of animals at two different points, one for visceral organs (e.g., lung, liver, heart, and kidney) and another for carcasses, by two OVIs following the stipulated steps in Commission Implementing Regulation (EU) no. 2019/627 [19]. Within this procedure, the concise macroscopic anatomical–pathological examination of the edible parts of animals included four standard procedures as follows: visual examination, palpation, olfaction, and performance of the necessary incisions, complemented by laboratory analyses for residues (e.g., detection of antimicrobials), to limit the chemical hazards to the consumer. Visual inspection aimed to evidence the abnormalities (e.g., presence of abscesses, cysts, or nodules) on the surface of muscle tissues and organs, besides their general appearance in color, dimension, volume, shape, or smoothness. The palpation, undertaken via touch and pressure to evidence irregularities, targeted the appreciation of elasticity, friability, temperature, humidity, hardness, stiffness, and dryness of the structures of edible parts of the animals. The necessary incisions, undertaken in agreement with the EU regulation requirements [18,19], were employed on the tongue, heart, and various lymph nodes to evidence inflammatory or undetectable pathological conditions (e.g., parasitic cysts or deep parenchymal lesions) via visual examination and palpation.

The OVIs interpreted and grouped the recorded pathological findings as previously indicated by Gracey [22] and Vallant [23]. In the case of carcasses, complete confiscations were associated with the occurrence of generalized abnormalities, improper pH, foreign smell, or non-compliant laboratory results; in the case of the presence of abnormalities limited to a certain area (e.g., abscesses, fecal contaminations, anomalies, and dystrophies), the damaged parts were trimmed, with further processing of the resulting parts suitable for human consumption. Regarding the edible viscera, only those that were completely free of lesions were admitted for human consumption. Furthermore, according to the policy of the abattoir in the case of kidneys, if one of them presented any type of abnormality, both were confiscated, as well as the spleen together with the gastrointestinal mass.

During examinations, the recorded anatomical–pathological findings for the slaughtered animals were registered into the VetScore^®^ (IMWT, GmbH, München, Germany) standardized online intranet platform of the slaughterhouse. The archived data were extracted and processed during manuscript preparation.

### 2.3. Estimation of Financial Loss

Several variables were included in the estimation of direct financial losses due to the confiscation of carcasses and offal, namely the number of seized carcasses (totally or partially) and organs, the average weight of the edible parts of animals (lung = 1.7 kg, liver = 1.8 kg, kidney = 0.15 kg, heart = 0.4 kg, and carcass = 110 kg) [24], and the commercial price of one kilogram of product (in EUR, lung = 2.2, liver = 2.1, kidney = 2.2, heart = 2.4, carcass = 2.8) established by the slaughterhouse for retail units. It is important to mention that for the partially confiscated carcasses, the computed average loss, based on the available statistical data in the slaughterhouse database, was established at 2.0%. This percentage was computed considering the losses due to carcass weight reduction during the study period expressed in kg, multiplied by 100, and divided with the expected total carcass weight without condemnations, based on the 110 kg average carcass weight of an animal. The estimation of the total economic losses due to confiscations was expressed in percentages, representing the summation of the computed losses for carcasses and each type of offal, reported to the total achievable net income, and without condemnations.

### 2.4. Statistical Analysis

Statistical analysis was performed using a commercially available software program (IBM, SPSS Advanced Statistics, 23.0, IBM Corp., Armonk, NY, USA). Differences in the recorded variables were analyzed using the one-way ANOVA test. Significance was assigned at *p* < 0.05.

## 3. Results

### 3.1. Causes of Carcass and Organ Condemnations

Table 1 presents the main causes of carcass and organ condemnations resulting after the slaughter of 307,866 pigs over two years (2021–2022) in a private abattoir located in Bavaria State, Germany. The resulting average value of animals slaughtered in one day was ~590.

Generally, the postmortem inspection results indicated that 0.17% (*n* = 522; 95% confidence interval = 0.16–0.19) of carcasses were confiscated, with dominant causes for pathological conditions in generalized diseases (e.g., septicemia, lymphadenitis, and erysipelas) (Table 1). Likewise, another 1.92% (*n* = 5900; 95% CI = 1.87–1.97) of carcasses was partially unfit for human consumption, especially due to abscesses, and traumatic and/or tail lesions (Table 1). Therefore, 2.09% (*n* = 6422; 95% CI = 2.04–2.14) of the examined carcasses presented pathological conditions or lesions.

Regarding the macroscopic examinations of organs, a total of 17.59% (*n* = 54,145; 95% CI = 17.46–17.72) expressed lesions or pathological conditions, with an extremely variable condemnation rate amongst the examined categories. Thus, 14.71% (*n* = 45,290; 95% CI = 14.59–14.84), 1.94% (*n* = 5968; 95% CI = 1.89–1.99), 0.72% (*n* = 2213; 95% CI = 0.69–0.75), and 0.22% (*n* = 674; 95% CI = 0.2–0.24) of the examined liver, lung, kidney, and heart specimens, respectively, presented anatomical–pathological findings. The liver was the most frequently affected organ, with parasitic disorders (53.4%) (especially in the form of “milk spots” in ascaridiasis) being the most common issue; however, condemnations due to dystrophies/anomalies were also significant (45.4%). In the case of the lungs, the suspected bacterial/viral and parasitic infections were more frequently encountered than circulatory disorders (63.5% vs. 36.5%). The main cause for kidney condemnation was the presence of dystrophies/anomalies (98.4%), while in the case of the heart, the occurrence of bacterial/viral infections (98.5%) caused the greatest damage (Table 1). Amongst the variables of the recorded pathological findings in the case of the liver, parasitic lesions were significantly more prevalent (*p* < 0.05) in 2021 compared with 2022, while dystrophies/anomalies were significantly more frequently accounted in 2022 compared with 2021 (Table 1, indicated with *). Furthermore, no statistically significant differences were found in the case of the recorded lesions of other organs and carcasses during the study years.

### 3.2. Financial Loss Estimations

In Table 2, the direct financial loss estimates are presented. For carcasses, a total loss of EUR 197,120 was estimated, representing 0.21% of the total achievable net revenue without condemnations, meaning EUR 94,822,728. Furthermore, the financial loss due to offal seizures was estimated at EUR 195,624.2, representing 6.95% of the total achievable net income, i.e., EUR 2,813,895.48. The biggest losses were attributed to liver condemnations (EUR 171,196.2), while the smallest losses were attributed to heart condemnations (EUR 647.1). Overall, within the investigated pig slaughterhouse, the total financial loss attributable to carcass and organ condemnations was EUR 392,744.2, which represents 0.40% of the total achievable net income (EUR 97,636,623.48).

## 4. Discussions

The results of the current study underline the usefulness of meat inspection at the slaughterhouse level, based on visual, palpatory, and incision techniques, as a source of information to detect and prevent notifiable diseases and zoonoses, identify production diseases, and offer useful feedback for veterinary practitioners regarding the occurrence of diseases in subclinical conditions at the farm level. However, meat inspection allows the evidence of gross lesions, but without offering information on the presence of major foodborne pathogens (e.g., pathogenic *E. coli* strains, *Salmonella* spp., *Campylobacter* spp., and *Listeria* spp.), chemical substances, or veterinary drugs as meat contaminants. These agents may be present at any stage of the slaughtering process when good manufacturing and hygiene practices are not present [13].

The postmortem inspection results of the present study indicated that 2.09% of the examined carcasses presented lesions or pathological conditions, resulting in 0.17% and 1.92% of total and partial condemnations, respectively. The number of observational studies focused on the causes of pig carcass condemnation is well represented in the scientific literature, reporting a variable overall condemnation rate from 0.03% [7] to 10.2% [10]. In most of them, subunit values were published. Thus, the recorded condemnation rates were 0.03% in Italy [7], 0.1% in Spain [11], 0.11% in Finland [25], 0.24% [5] in Portugal, 0.3% in Finland [26], 0.37% in Canada [27], and 0.57% in the Czech Republic [28]. Nonetheless, much higher confiscation rates were obtained in other studies conducted in Spain [8.5%; 6] and Brazil [10.2%; 10]. Furthermore, in a Ghanaian study, the reported total and partial condemnation rates were 1.72% and 15.64%, respectively [15]. The observed differences in the condemnation rates, reported in different studies, must be interpreted cautiously, considering that characteristics related to the study design (e.g., seasonal variations, data reporting, and abattoir characteristics), geo-climatic conditions of the study region, and the pig farming system can markedly influence the presented results [2]. Furthermore, the lack of harmonization in the terminology of meat inspection criteria can contribute to recorded differences.

Regarding the main causes of complete carcass condemnations, the presence of generalized pathological conditions (e.g., septicemia, cachexia, pyemia, lymphadenitis, and vertebral osteomyelitis) accounted for 62.6% (Table 1). In this regard, septicemia, a life-threatening phenomenon that means the invasion of the bloodstream by pathogenic bacteria, was involved in the total condemnation of 0.27% of pig carcasses in Italy [7]. Specifically, erysipelas, a common disease of pigs caused by *Erysipelothrix rhusiopathiae* and typically characterized by the presence of diamond-shaped skin lesions, acute septicemia, and/or polyarthritis, has also been involved in 0.77%, 1.20%, and 37.36% of carcass condemnations in Portugal [5], Spain [6], and Italy [7], respectively. Moreover, different condemnation rates were reported for cachexia in Portugal [1.79%; 5], Spain [28.9%; 6], and Italy [2.69%; 7], as well as for generalized pyemia, manifested with the widespread presence of abscesses, in Spain [38.4%; 6]. Furthermore, the presence of lymphadenitis, as a consequence of the occurrence of systematic diseases in pigs (e.g., infections produced by *Mycobacterium avium* complex, *Streptococcus* spp., or *Trueperella pyogenes*) [12] was reported as an important cause of total carcass condemnations in several countries (e.g., 22.7% in Portugal [5], 1.20% in Spain [11], 0.29% in Brazil [10], and 0.08% in the Czech Republic) [9]. Nevertheless, it is important to mention that, as has been observed in the present study, the involvement of vertebral osteomyelitis in whole carcass condemnations, having tail biting as a predisposing factor, has been previously mentioned in some investigations conducted in Portugal [5,9].

Secondly, the presence of abnormal changes during postmortem examinations and errors in the slaughtering process provides an important contribution to the complete carcass condemnations within the current research (Table 1). In this regard, other studies displayed similar results concerning condemnations in the case of the occurrence of pale soft and exudative meat [5], insufficient bleeding [11], generalized jaundice [3,5,6,7,11], and sensorial alterations [9]. The appearance of pale, soft, and exudative meat may be related to improper pre-slaughter handling of animals [29], while insufficient bleeding could occur in case of agonic aspiration of blood due to inadequate exsanguination [14]. Condemnations due to generalized jaundice, meaning the discoloration in yellow of tissues and blood plasma due to accumulation of bile pigments, suggests the need for a careful risk assessment, considering the possible presence of toxic or bacteriological/viral systemic infections (e.g., copper, mycotoxins, *Mycoplasma suis*, or porcine circovirus type 2) [3,30]. The presence of pathological conditions due to preformed volatile organic and odor compounds in the meat [31] highlights the importance of sensorial analysis during postmortem inspection.

In line with the obtained results in the present survey, abscesses [3,5,7,10], scabies [10], traumatic lesions [7], adherences [10], tail lesions [6], and contamination by eviscerating leaking [10] have been mentioned as causes of partial carcass condemnations in several other studies. In this case, the affected portions were trimmed, and the remaining parts were admitted for human consumption.

The recorded relevant pathological findings in the case of the examined offal underline the importance of animal health control at the slaughterhouse level, as a useful tool for the knowledge and proper management of animal diseases at the primary production stage [32]. In this regard, a lower condemnation rate was observed for pulmonary problems compared to those recorded at the liver level (Table 1). Industrial pig farming under controlled environmental conditions (e.g., temperature, humidity, and ventilation) could explain these findings [2,33]. The main causes of liver condemnation were related to parasitism such as hydatidosis and ascariasis, reflecting deficiencies in deworming programs of the production units, but the recorded dystrophies/anomalies, most of them derived from chronic parasitism (Table 1), also had an important contribution. These results are in line with those reported elsewhere [6,13,34], and confirmed that *Ascaris* larval migration, producing the so-called “milk spots” pathological condition, generates significant losses in the pig breeding industry. Furthermore, pneumonia, related to bacterial and viral infections (e.g., *Trueperella* spp., *Pasteurella* spp., *Mycoplasma hyorhinis*, *Glaesserella parasuis*, *Clostridium* spp., and *Streptococcus suis*) [23], and the presence of abscesses and lungworms showed dominant occurrence towards circulatory disorders (e.g., congestion, emphysema, edema, or hemorrhages) as causes of pulmonary condemnations (Table 1). These findings have been previously confirmed in other investigations [5,6,10,27], indicating that respiratory pathological conditions, as a common cause of lung condemnations, remain an key concern for swine herds worldwide [35]. Remarkably, the encountered consequences of bacterial and/or viral infections, as the principal cause of heart condemnations in the present study, usually occur secondary to respiratory problems [36]. It is important to mention, in the case of the carcass, liver, and heart specimens, the occurrence of fecal contamination as a consequence of human error during slaughtering. Thus, the employment of highly professionally trained and experienced butchers with a good ability to concentrate during slaughtering can be considered an important factor in reducing financial losses. Likewise, the occurrence of congenital cysts and hydronephrosis, consisting of the distension of the renal pelvis and calyces with urine due to the obstruction of the urinary outflow tract, leading to renal failure, were accounted as the main causes of kidney condemnations. The evidence of this gross lesion can be attributed to a nutritional imbalance between the ratio of calcium and phosphorus in the diet [37]. Last, but not least, it is important to mention that the observed fecal contamination at the carcass as well as organ level underline the importance of the development of control systems such as Hazard Analysis Critical Control Points (HACCPs) to decrease the microbiological risk (*Salmonella* spp., *Campylobacter*, and *Yersinia* spp.) during slaughtering, and especially within the evisceration process [13]. In the case of the liver, the recorded significantly higher prevalence values for parasitic lesions, as well as for dystrophies/anomalies in different years compared to the others, suggest a seasonality pattern in the occurrence of these pathological conditions. This can be related to feeding and/or environmental factors (e.g., humidity, temperature, and suitable ground conditions for *Ascaris suis* eggs), as has been previously observed by Elbers et al. [38], but further investigations, based on the data taken from several years [13], are still necessary to strengthen this hypothesis.

Out of the estimated total monetary losses (EUR 392,744.2) in the present investigation over the two-year study period, resulting from the slaughtering of 307,866 pigs and representing 0.40% of the total achievable net revenue without condemnations (EUR 97,636,623.24), 50.19% (EUR 197,120) were attributable to carcass condemnations and 49.81% (EUR 195,624.2) to organ condemnations (Table 2). However, in the case of the partially confiscated carcasses, it is important to mention that caution should be taken in interpreting the financial losses because the estimations do not express the different commercial values of edible parts of the animals (e.g., ribs, leg, loin, and ham). In addition, the interpretation and classification of the presented pathological findings, especially those leading to confiscations and economic losses, are strongly related to the experience, mental concentration, professional training, knowledge, and motivation of OVIs involved in meat inspection [32]. The number of studies that attempted to quantify the financial losses due to carcass and offal condemnations of the slaughtered pigs is scarce and outdated, and the presented data are inconsistent. Thus, in a relevant study conducted in Costa Rica by Mateus-Vargas et al. [13] between 2007 and 2009, from a total of 526,843 slaughtered pigs, the accounted economic losses from the rejections of whole viscera were USD 254,048.1. Similarly to our results, the greatest losses were accounted for in the case of the liver condemnations (USD 208,167.8), followed by kidney condemnations (USD 35,744) and heart condemnations (USD 10,136.3). In another study, the economic implications resulting from carcass and viscera rejection in a population of 2,959,607 pigs admitted for slaughtering between 1984 and 1986 in Singapore accounted for USD 5,270,000, meaning USD 1.78 per pig admitted [8]. Furthermore, in Ghana, from August to December 2017, the postmortem examination of 1221 pigs resulted in a USD 21,745.89 financial loss due to carcass and organ seizures [15]. In addition, in an investigation conducted by Hill and Jones [16], the combined annual loss from meat and viscera rejection of 510,880 slaughtered pigs was GBP 149,277. These results emphasize the fact that, despite efforts to increase the biological efficiency of pigs in terms of growth and reproduction in recent decades, the improper management of disease during breeding can generate significant losses.

## 5. Conclusions

The description and analysis of the collected data on carcass and organ condemnations of slaughtered pigs in the present study indicate some relevant considerations. Thus, the occurrence of generalized diseases, as the main reason for the whole rejection of carcasses, further stimulates the necessity of the introduction of the etiological diagnosis of pathological conditions during routine meat inspection, in order to complete and improve the present knowledge of the burden of swine pathology at the abattoir level. Furthermore, in the case of the organs, the highest number of pathological conditions was registered for the liver, especially due to the occurrence of parasitic disease, highlighting the importance of deworming programs during the production stage. The survey also showed that the recorded total amounts of carcass and viscera condemnations each had a similar financial impact. Altogether, the results of this study highlight the importance of carcass and viscera condemnation data at the slaughterhouse level to implement effective control measures for animal disease and limit public health hazard propagation via the food chain.

## Figures and Tables

**Table 1:** Overview on the distribution of main causes of carcass and organ condemnation in the 307,866 pigs slaughtered during the study period (2021–2022).

Causes of Carcass and Organ Condemnations	Year				Total Examined = 307,866		Total Number of Condemnations (%)
	2021 (*n* = 173,243)		2022 (*n* = 134,623)				
	Number	Percent	Number	Percent	Number	Percent	
Carcass with pathological conditions (*n* = 6422; 2.09%)							522 (8.12)
Total condemnations							
generalized diseases (septicemia, pyemia, cachexia, lymphadenitis, vertebral osteomyelitis, etc.)	192	0.11	135	0.1	327	0.1	
errors in the slaughtering process and abnormal changes (pale soft and exudative meat, febrile meat, insufficient bleeding, sensorial changes, and generalized jaundice)	39	0.02%	59	0.04	98	0.03	
pleurisy, polyarthritis,	26	0.015	39	0.03	65	0.02	
peritonitis, polyserositis	17	0.01	15	0.01	32	0.01	
Partial condemnations							5900 (91.88)
abscesses, scabies, traumatic lesions, adherences, tail lesions	2071	1.2	2104	1.6	4175	1.4	
contamination by eviscerating leaking	481	0.3	558	0.4	1039	0.34	
dystrophies/anomalies	317	0.18	369	0.27	686	0.2	
Organs with abnormalities (*n* = 54,145; 17.59%)							
Lung							5968 (1.94)
suspected bacterial/viral and parasitic infections (pneumonia, pleurisy, abscesses, lungworms, etc.)	2188	1.3	1602	1.2	3790	1.23	
circulatory disorders (congestion, edema, emphysema, hemorrhages, etc.)	1393	0.8	785	0.6	2178	0.7	
Liver							45,290 (14.71)
parasitic (hydatidosis, ascaridiasis, etc.)	13,970	8.1 *	10,260	7.6	24,230	7.9	
suspected bacterial/viral	40	0.02	21	0.015	61	0.02	
contaminated feces	259	0.15	159	0.12	418	0.14	
dystrophies/anomalies (cirrhosis, icterus, hepatomegaly, fatty degeneration, etc.)	11,108	6.4	9473	7.0 *	20,581	6.7	
Kidney							2213 (0.72)
suspected bacterial/viral	20	0.01	16	0.01	36	0.01	
dystrophies/anomalies (hydronephrosis, congenital cysts, etc.)	1188	0.7	989	0.7	2177	0.7	
Heart							674 (0.22)
suspected bacterial/viral (abscesses, pericarditis, endocarditis, etc.)	365	0.21	299	0.22	664	0.21	
contaminated feces	6	0.003	4	0.003	10	0.003	

Legend: * statistically significant differences (*p* < 0.05) between the number of condemned organs recorded over the study period.

**Table 2:** Estimated financial losses due to the condemnation of the carcasses and organs resulting from the 307,866 slaughtered pigs (expressed in EUR).

Type of Carcass/Organs	No. of Condemned Carcasses/Organs	Average Weight (kg)	Total Condemned Weight (kg)	Price/kg (EUR)	Financial Losses (EUR)	Total Estimated Loss (%)/Estimated Revenue (EUR)
Carcass						197,120 (0.21)/94,822,728
totally	522	110	57,420	2.8	160,776	
partially	5900	110	12,980 *	2.8	36,344	
Organ						195,624.2 (6.95)/2,813,895.48
Liver	45,290	1.8	81,522	2.1	171,196.2	
Lung	5968	1.7	10,145.6	2.2	22,320.3	
Heart	674	0.4	269.6	2.4	647.1	
Kidney	2213	0.15 (×2)	663.9	2.2	1460.6	
Total						392,744.2 (0.40)/97,636,623.48

Legend: *—the value represents 2.0% of the total amount of condemnations expressed in kg.

## Data Availability

The data are contained within the article.

## References

[1] Hoque M., Mondal S., Adusumilli S. (2022). Sustainable Livestock Production and Food Security. Emerging Issues in Climate Smart Livestock Production.

[2] García-Díez J., Saraiva S., Moura D., Grispoldi L., Cenci-Goga B.T., Saraiva C. (2023). The importance of the slaughterhouse in surveilling animal and public health: A systematic review. Vet. Sci..

[3] Rosamilia A., Galletti G., Benedetti S., Guarnieri C., Luppi A., Capezzuto S., Tamba M., Merialdi G., Marruchella G. (2023). Condemnation of Porcine Carcasses: A Two-Year Long Survey in an Italian High-Throughput Slaughterhouse. Vet. Sci..

[4] Mateos G.G., Corrales N.L., Talegón G., Aguirre L. (2024). Invited Review—Pig meat production in the European Union-27: Current status, challenges, and future trends. Anim. Biosci..

[5] García-Díez J., Coelho A.C. (2014). Causes and factors related to pig carcass condemnation. Vet. Med..

[6] Martínez J., Jaro P.J., Aduriz G., Gómez E.A., Peris B., Corpa J.M. (2007). Carcass condemnation causes of growth retarded pigs at slaughter. Vet. J..

[7] Guardone L., Vitali A., Fratini F., Pardini S., Cenci Goga B.T., Nucera D., Armani A. (2020). A retrospective study after 10 years (2010–2019) of meat inspection activity in a domestic swine abattoir in Tuscany: The slaughterhouse as an epidemiological observatory. Animals.

[8] Tiong C.K., Bin C.S. (1989). Abattoir condemnation of pigs and its economic implications in Singapore. Br. Vet. J..

[9] Vieira-Pinto M., Azevedo J., Poeta P., Pires I., Ellebroek L., Lopes R., Veloso M., Alban L. (2020). Classification of vertebral osteomyelitis and associated judgment applied during post-mortem inspection of swine carcasses in Portugal. Foods.

[10] Coldebella A., Kich J.D., Albuquerque E.R., Buosi R.J. (2017). Reports of Brazilian federal meat inspection system in swine slaughterhouses. International Symposium on the Epidemiology and Control of Biological, Chemical and Physical Hazards in Pig and Pork, 12, Foz do Lguaçu.

[11] Sánchez P., Pallarés F.J., Gómez M.A., Bernabé A., Gómez S., Seva J. (2018). Importance of the knowledge of pathological processes for risk-based inspection in pig slaughterhouses (Study of 2002 to 2016). Asian-Australas. J. An. Sci..

[12] Cardoso-Toset F., Gómez-Laguna J., Gómez-Gascón L., Rodríguez-Gómez I.M., Galán-Relaño A., Carrasco L., Tarrdas C., Vela A.I., Luque I. (2020). Histopathological and microbiological study of porcine lymphadenitis: Contributions to diagnosis and control of the disease. Porc. Health Manag..

[13] Mateus-Vargas R.H., Jimenez-Loaiza E.M., Alfaro-Zuniga C.E., Passos-Pequeno A. (2011). Analysis of the most common causes of viscera condemnation in pigs (liver, kidney, heart), in a slaughterhouse of Costa Rica, and its economical implication. Arch. Leb..

[14] Ferreira M.F., Fàbrega E., Pires I., Vieira-Pinto M.M. (2023). Agonic Aspiration of Blood: Not Useful as an Animal-Based Indicator of Electrical Stunning Ineffectiveness in Pigs (*Sus scrofa domesticus*). Animals.

[15] Atawalna J., Yeboah D.A., Ovoro M.R. (2019). Causes of carcass condemnation and its associated financial losses in slaughtered pigs at the Kumasi abattoir Company Limited. J. Dairy Vet. Sci..

[16] Hill J.R., Jones J.E.T. (1984). An investigation of the causes and of the financial loss of rejection of pig carcasses and viscera unfit for human consumption. I. Studies at one abattoir. Br. Vet. J..

[17] (2023). Bavarian State Office for Statistics. https://www.tridge.com/news/the-pig-population-in-bavaria-is-clearly-declining.

[18] European Parliament (2017). Council of the European Union. Regulation (EU) 2017/625 of the European Parliament and of the Council of 15 March 2017 on official controls and other official activities performed to ensure the application of food and feed law, rules on animal health and welfare, plant health and plant protection products, amending Regulations (EC) No 999/2001, (EC) No 396/2005, (EC) No 1069/2009, (EC) No 1107/2009, (EU) No 1151/2012, (EU) No 652/2014, (EU) 2016/429 and (EU) 2016/2031 of the European Parliament and of the Council, Council Regulations (EC) No 1/2005 and (EC) No 1099/2009 and Council Directives 98/58/EC, 1999/74/EC, 2007/43/EC, 2008/119/EC and 2008/120/EC, and repealing Regulations (EC) No 854/2004 and (EC) No 882/2004 of the European Parliament and of the Council, Council Directives 89/608/EEC, 89/662/EEC, 90/425/EEC, 91/496/EEC, 96/23/EC, 96/93/EC and 97/78/EC and Council Decision 92/438/EEC (Official Controls Regulation). Off. J. Eur. Union.

[19] European Commission (2019). Commission Implementing Regulation (EU) 2019/627 of 15 March 2019 laying down uniform practical arrangements for the performance of official controls on products of animal origin intended for human consumption in accordance with Regulation (EU) 2017/625 of the European Parliament and of the Council and amending Commission Regulation (EC) No 2074/2005 as regards official controls. Off. J. Eur. Union.

[20] Lahti P., Soini J., Ninios T., Lunden J., Korkeala H., Fredriksson-Ahomaa M. (2014). Ante-mortem inspection. Meat Inspection and Control in the Slaughterhouse.

[21] Regulation (EC) No 852/2004 of the European Parliament and of the Council of 29 April 2004 on the Hygiene of Foodstuffs. http://data.europa.eu/eli/reg/2004/852/oj.

[22] Gracey J.F. (1986). Meat Hygiene.

[23] Vallant A. (2010). Taschenatlas Schlachttierkörper-Pathologie bei Rind Und Schwein.

[24] Barone R. (2009). Anatomie Comparée des Mammifères Domestiques, Tome 3, Splanchnlogie I, Appareil Digestif.

[25] Heinonen M., Bergman P., Fredriksson-Ahomaa M., Virtala A.M., Munsterhjelm C., Valros A., Oliviero C., Peltoniemi O., Hälli O. (2018). Sow mortality is associated with meat inspection findings. Livest. Sci..

[26] Thompson K., Grant Maxie M. (2006). Bones and joints. Jubb, Kenedy and Palmer’s Pathology of Domestic Animals.

[27] Amezcua R., Pearl D.L., Martinez A., Friendship R.M. (2011). Patterns of condemnation rates in swine from a federally inspected abattoir in relation to disease outbreak information in Ontario (2005–2007). Can. Vet. J..

[28] Kozak A., Vecerek V., Chloupek P., Tremlova B., Malena M. (2003). Veterinary meat inspection of pig carcasses in the Czech Republic during the period of 1995–2002. Vet. Med..

[29] Ogawa N.N., Silva G.L., Barbon A.P.A.D.C., Flaiban K.K.M.D.C., Silva C.A.D., Rocha L.M., Bridi A.M. (2024). Animal Welfare Assessment and Meat Quality through Assessment of Stress Biomarkers in Fattening Pigs with and without Visible Damage during Slaughter. Animals.

[30] Graage R., Saura Martinez H., Klausmann S., Kubacki J., Kümmerlen D. (2020). Intrahepatic icterus in pigs: Rare clinical sign in porcine circovirus type 2 systemic disease. Vet. Rec. Case Rep..

[31] Bleicher J., Ebner E.E., Bak K.H. (2022). Formation and Analysis of Volatile and Odor Compounds in Meat—A Review. Molecules.

[32] Ciui S., Morar A., Tîrziu E., Herman V., Ban-Cucerzan A., Popa S.A., Morar D., Imre M., Olariu-Jurca A., Imre K. (2023). Causes of Post-Mortem Carcass and Organ Condemnations and Economic Loss Assessment in a Cattle Slaughterhouse. Animals.

[33] Stärk K.D. (2000). Epidemiological investigation of the influence of environmental risk factors on respiratory diseases in swine—A literature review. Vet. J..

[34] Villá D.M., Pérez N.A.I., Parga E.P., Escobar Y.T. (2017). Causes of Liver, Kidney and Heart Condemnation at Swine Slaughterhouse in Ciego de Avila, Cuba. Rev. Prod. Anim..

[35] Przyborowska-Zhalniarovich P., Maes D., Otrocka-Domagała I., Paździor-Czapula K., Wiszniewska-Łaszczych A., Sołtysiuk M. (2023). Association between Enzootic Pneumonia-like Lung Lesions and Carcass Quality and Meat pH Value in Slaughter Pigs. Animals.

[36] Petri F.A.M., Ferreira G.C., Arruda L.P., Malcher C.S., Storino G.Y., Almeida H.M.d.S., Sonalio K., Silva D.G.d., Oliveira L.G.d. (2023). Associations between Pleurisy and the Main Bacterial Pathogens of the Porcine Respiratory Diseases Complex (PRDC). Animals.

[37] Lorenzett M.P., Cruz R.A.S., Cecco B.S., Schwertz C.I., Hammerschmitt M.E., Schu D.T., Driemeier D., Pavarini S.P. (2019). Obstructive urolithiasis in growing-finishing pigs. Pesqui. Veterinária Bras..

[38] Elbers A.R.W., Tielen M.J.M., Snijders J.M.A., Cromwijk W.A.J., Hunneman W.A., Epidemiological studies on lesions in finishing pigs in the Netherlands (1992). I prevalence, seasonality and interrelationship. Prev. Vet. Med..

